# Intratumoral injection of anlotinib hydrogel enhances antitumor effects and reduces toxicity in mouse model of lung cancer

**DOI:** 10.1080/10717544.2020.1837292

**Published:** 2020-10-29

**Authors:** Qin Gao, Shan Tang, Han Chen, Hui Chen, XiaoJie Li, YiQing Jiang, ShaoZhi Fu, Sheng Lin

**Affiliations:** Department of Oncology, Affiliated Hospital of Southwest Medical University, Luzhou, China

**Keywords:** Anlotinib, hydrogel, anti-angiogenesis, Lewis lung cancer, hyaluronic acid–tyramine

## Abstract

This study was conducted to determine the antitumor effects and ability of an anlotinib (AL) hydrogel (AL–HA–Tyr) to reduce toxicity in a mouse model of Lewis lung cancer (LLC). We constructed a drug carrier system for AL, verified its effectiveness and systemic safety, and provided a preliminary experimental foundation for clinical carrier transformation. AL–HA–Tyr was prepared by encapsulating AL with hyaluronic acid–tyramine (HA–Tyr) conjugates. Colony and tube formation assays showed that AL–HA–Tyr restrained the proliferation of human umbilical vein endothelial cells (HUVECs) and LLC cells, respectively, *in vitro*, and that AL exerted significant anti-angiogenesis and anti-tumor effects. The invasion and migration of HUVECs and LLC cells were efficiently suppressed by AL according to transwell assays. HUVEC and LLC cell-cycle and apoptosis analysis clarified the direct anti-tumor effects of AL–HA–Tyr. Mice engrafted with LLC cells *in vivo* were administered oral saline, oral AL, or an intratumoral injection of HA–Tyr or AL–HA–Tyr. The results showed that AL–HA–Tyr obviously reduced visceral toxicity and decreased Ki67 and VEGF-A expression in tumor cells compared with AL. Furthermore, AL–HA–Tyr significantly prolonged the survival of tumor-bearing mice. Overall, AL–HA–Tyr enhanced antitumor effects and reduced toxicity in the LLC model. It provided a foundation for the clinical transformation of drug carrier systems.

## 1. Introduction

Lung cancer is the leading cause of cancer-related deaths worldwide (Bray et al., 2018). Although comprehensive therapies and novel clinical drugs have been developed, therapeutic outcomes remain poor (Torre et al., 2016). Targeted antiangiogenic drugs are important modalities for tumor therapy, as angiogenesis provides nutritional support required for tumorigenesis and tumor progression (Viallard & Larrivee, 2017). Anlotinib (AL) hydrochloride is a promising novel, small-molecule, multi-target, tyrosine (Tyr) kinase inhibitor that has been approved as a treatment option when multiline therapies against advanced solid tumors fail (Shen et al., 2018). Like other antiangiogenic drugs, the most prevalent adverse event of AL is hypertension (Si et al., 2019), which can lead to interrupted treatment and threaten life. Thus, a more effective drug delivery system with lower toxicity and good biological compatibility is highly desirable.

Injectable hydrogels based on naturally occurring polymers have gained considerable attention because they are inherently non-inflammatory, biocompatible, non-immunogenic, and biodegradable (Bermejo-Velasco et al., 2018). Hyaluronic acid (HA) and its derivatives have been widely used in the clinic and applied to transport and release incorporated proteins and anticancer drugs (Zhang et al., 2019). Conjugates of HA are tunable and have better therapeutic efficacy than free anticancer drugs (Wang et al., 2019; Zhong et al., 2019).

The diagnostic accuracy of peripheral lung cancer is improved by CT-guided percutaneous lung biopsy. This has important clinical value for injecting intratumoral agents in a bolus. Therefore, we investigated whether AL could be encapsulated within a HA hydrogel to enable sustained release. We examined the validity and systemic safety of the hydrogel, analyzed its antiangiogenic effects on human umbilical vein endothelial cells (HUVECs) and antitumor effects in Lewis lung cancer (LLC) models, and provided a foundation for the clinical transformation of drug carrier systems.

## 2. Materials and methods

### 2.1. Reagents

AL (dihydrochloride form; purity > 99%) (Jiangsu Chia-tai Tianqing Pharmaceutical Co., Ltd. (Nanjing, China) was dissolved in double-distilled water to various concentrations for oral and intra-tumor administration in mice. Sodium HA (purity >95%, MW, 90 kDa), tyramine hydrochloride (Tyr·HCl), *N*-hydroxysuccinimide (NHS), 1-ethyl-3-(3-dimethylaminopropyl)-carbodiimide hydrochloride (EDC·HCl), hydrogen peroxide (H_2_O_2_, 30 wt.%), horseradish peroxidase (HRP, 100 U/mg), and bovine testicular hyaluronidase were purchased from MeiLun Co., Ltd. (Dalian, China). Dimethyl sulfoxide and crystal violet were purchased from Kelong Co., Ltd. (Chengdu, China). Polyclonal antibodies against Ki-67 and VEGF-A were purchased from Bioworld Technology Co., Ltd. (Nanjing, China). The Cell Cycle and Apoptosis Analysis Kit and YF647A-Annexin V and PI Apoptosis Kit were purchased from US Everbright, Inc. (Nanjing, China).

### 2.2. Cell lines

LLC cells and HUVECs were obtained from the experimental laboratory of Southwest Medical University (Luzhou, China) and cultured in Dulbecco’s Modified Eagle Medium (DMEM; HyClone, Logan, UT) supplemented with 10% fetal bovine serum (HyClone, Logan, UT), 0.1 mg/mL streptomycin, and 100 U/mL penicillin in a humidified 5% CO_2_ atmosphere at 37 °C.

### 2.3. Preparation and biochemical characteristics of AL–HA–Tyr

Conjugates of hyaluronic acid–tyramine (HA–Tyr) were prepared as follows (Lee et al., 2008). Briefly, HA (1 g) and Tyr·HCl (202 mg) were dissolved in 100 mL of distilled water. Thereafter, EDC·HCl (479 mg) and NHS (290 mg) were added and the pH was adjusted to 7.0 with 0.1 M NaOH overnight. Next day, the mixture was placed in dialysis bags (molecular weight cutoff = 1000 Da) and dialyzed against 100 mM sodium chloride, followed by ethanol (25%) and distilled water for one day each. The dialysate was lyophilized and analyzed by proton nuclear magnetic resonance (^1^H NMR). The gelation time of the hydrogel was judged by continuously tilting a tube after HA–Tyr (1.0 wt.%), HRP, and H_2_O_2_ were dissolved in distilled water at 37 °C. The gelling point of the hydrogel was defined as the absence of liquid flow within 30 s of inverting the tube. We prepared AL–HA–Tyr by dissolving HA–Tyr (1.0 wt.%) and AL (different concentrations) in distilled water, and then adding HRP (50 U/mL) and H_2_O_2_ (20 mM) at 25 °C. *In vitro*, no enzyme can catalyze hydrogel degradation and drug release. Therefore, different amounts of enzymes were added. AL–HA–Tyr-5 and AL–HA–Tyr-25 indicate that 5 and 25 U/mL hyaluronidases were added to the AL–HA–Tyr, respectively.

### 2.4. Analysis of drug release *in vitro* by high-performance liquid chromatography (HPLC)

Solutions of AL and AL–HA–Tyr-25 (2 mL each) were loaded into separate dialysis bags (molecular weight cutoff = 1000 Da), immersed in 40 mL of double distilled water with or without 25 U/mL of hyaluronidase, and shaken at 80 r/min in a water bath using a thermostatic oscillator at 37 °C. Samples (2 mL) were removed at predetermined intervals and replaced with the same volume of fresh medium. The supernatants were stored at −20 °C, and 20 μL samples were analyzed by HPLC (Agilent Technologies, Santa Clara, CA) using a reverse phase C18 column (4.6 × 50 mm; particle size, 3.5 mm) at a constant temperature of 25 °C. The mobile phases A (CH_3_OH in 0.1% TFA, v/v) and B (HPLC-grade water in 0.1% TFA, v/v) were ultrasonically degassed before use. The optimal gradient elution program was as follows: 0 min, 10% A; 0–15 min, linear 10–100% A; 20 min, 100% A. The post time was 3.0 min for equilibration of the column and the total run time was 23.0 min. The flow rate was 1.0 mL/min and detection wavelength was 250 nm.

### 2.5. Colony formation assays

Single-cell suspensions were seeded into six-well plates (500 cells/well). After adhering to the walls, the cells were incubated with free AL (0, 1, 2, 5, 10, and 20 µM) or AL–HA–Tyr-5 (0, 1, 2, 5, 10, and 20 µM AL–HA–Tyr + 5 U/mL hyaluronidase) and NS, HA–Tyr, AL, AL + HA–Tyr or AL–HA–Tyr + lysozyme-5 for 24 h and further incubated at 37 °C for 10 days. Visible colonies were washed with PBS, fixed with 4% methanol, and stained with 0.05% crystal violet. The plates were gently washed and photographed. Visible colonies of >50 cells were counted, and the rate (%) of colony inhibition was calculated as:

Inhibition rate (%) = 1 − colony counts/colony counts in control × 100%.

### 2.6. Endothelial cell tube formation assays

We coated 96-well plates with cold Matrigel^®^ (60 μL/well) at 37 °C for 30 min, and implanted HUVECs (2 × 10^4^ cells/well) on the surface of the matrix. The mixed supernatant media (control (DMEM)), HA–Tyr (1.0 wt.%), AL (5 μM), AL–HA–Tyr (AL: 5 μM, HA–Tyr: 1.0 wt.%), and AL–HA–Tyr + lysozyme-5 (AL: 5 μM, HA–Tyr: 1.0 wt.%, lysozyme: 5 U/mL) were added to each well and examined by microscopy (magnification, ×100) 6 h later. Three images were randomly selected and analyzed using ImageJ software (NIH, Bethesda, MD).

### 2.7. Invasion and migration assays

Cell invasion and migration were assayed as described previously (Yang et al., 2019). Polycarbonate 6.5-mm transwells with 8.0-μm pore polycarbonate membrane inserts (Corning, Inc., Corning, NY) were used with and without 90 μL of diluted (Matrigel:DMEM = 1:8) Matrigel^®^ (Corning, Inc., Corning, NY) for invasion and migration assays, respectively. Briefly, cell suspensions (4 × 10^4^) in 200 μL of serum-free medium were added to the upper chambers of 24-well transwell^®^ culture plates containing 10% fetal bovine serum. The various release solutions (control (DMEM)), HA–Tyr (1.0 wt.%), AL (5 μM), AL–HA–Tyr (AL: 5 μM, HA–Tyr: 1.0 wt.%), and AL–HA–Tyr + lysozyme-5 (AL: 5 μM, HA–Tyr: 1.0 wt.%, lysozyme: 5 U/mL) were placed in the lower chamber and incubated at 37 °C for 24 h. The chambers were then fixed with 4% methanol and stained with 0.05% crystal violet. Finally, the cells were gently removed from the upper surface of chambers using cotton swabs, gently washed with PBS, and air-dried. Finally, the cells were photographed using an optical inverted microscope (Olympus IX73 microscope, Tokyo, Japan) in six random fields (magnification, ×200) and counted with ImageJ software (NIH, Bethesda, MD).

### 2.8. Cell apoptosis and cell-cycle analysis

Apoptosis was analyzed with an YF647A-AnnexinV and propidium iodide (PI) apoptosis kit according to the manufacturer’s instruction. Briefly, the HUVECs and LLC cells were inoculated into six-well plates at a density of 5.0 × 10^4^ cells/well. The cells were treated with NS, HA–Tyr, AL, or AL–HA–Tyr for 24 h. After digestion with trypsin and centrifugation for 5 min at 300×*g*, the cells were washed with cold PBS and centrifuged again. Complete medium was added and incubated at 37 °C for 30 min, and the cells were washed twice and centrifuged for 5 min at 300×*g*. The cells (1.0 × 10^5^) were collected and re-selected with 100 μL of 1× buffer. Next, 5 μL PI and 5 μL YF647A-Annexin were added to and incubated for 15 min in the dark. The cells were analyzed by DxFlex flow cytometry (Beckman Coulter, Brea, CA). For cell cycle analysis, the treated cells were washed with cold PBS and centrifuged for 5 min at 1000×*g*. The cells were suspended in 1 mL 75% cold ethanol and fixed overnight at −20 °C. After centrifugation, the cells were washed, centrifuged, and then incubated in the dark with PI and RNaseA staining buffers for 30 min. The stained cells were analyzed by DxFlex flow cytometry.

### 2.9. Establishment and treatment of mouse models

The Chongqing Tengxing Experimental Animal Center provided 48 female C57BL/6J mice aged 4–6 weeks. All animal experiments were approved by the Institutional Animal Southwest Medical Care and Use Committee (Luzhou, China), and performed in accordance with the Institutional Animal Care and Use Guidelines. Mouse models were established by injecting 100 μL of LLC cell suspensions (1.5 × 10^7^ cells/mL) into the dorsal side of the right foot of each mouse. Seven days later, the tumor volume reached 100–200 mm^3^, and tumor-bearing mice were randomly divided into four groups of 12. The mice were orally administered with 0.9% saline (control) daily or AL (3 mg/kg/day) or intratumorally injected with HA–Tyr (1.0 wt.%) or AL–HA–Tyr (3 mg/kg/day × 3) at four-day intervals for 14 days (Xie et al., 2018). The weight and tumor size were measured every two days. Tumor volume was calculated using a caliper as (length × width^2^)/2. Six mice per group were sacrificed at random after 14 days, and the tumors and organs were collected. The other six mice in each group were maintained until death to determine their survival rates.

### 2.10. Histopathology and immunohistochemistry

The harvested tumors and organs were fixed with formalin, embedded in paraffin, and cut into 4-μm-thick sections for histopathological and immunohistochemical evaluation. Sections of the heart, lungs, liver, kidneys, and spleen were stained with hematoxylin and eosin (HE). Tumor samples were immunostained with Ki-67 and VEGF-A antibodies. Images were captured using an optical inverted microscope, and six regions were randomly selected for analysis using Image-ProPlus 6.0 software (Media Cybernetics, Rockville, MD).

### 2.11. Statistical analysis

Data were analyzed by unpaired two-tailed *t*-tests and expressed as the means ± standard deviation (SD). Survival was assessed using Kaplan–Meier’s curves. All data were analyzed using SPSS version 17.0 software (SPSS, Inc., Chicago, IL). Results with *p*<.05 were considered as statistically significant.

## 3. Results

### 3.1. Preparation and biochemical characteristics of AL–HA–Tyr

**Figure 1.**
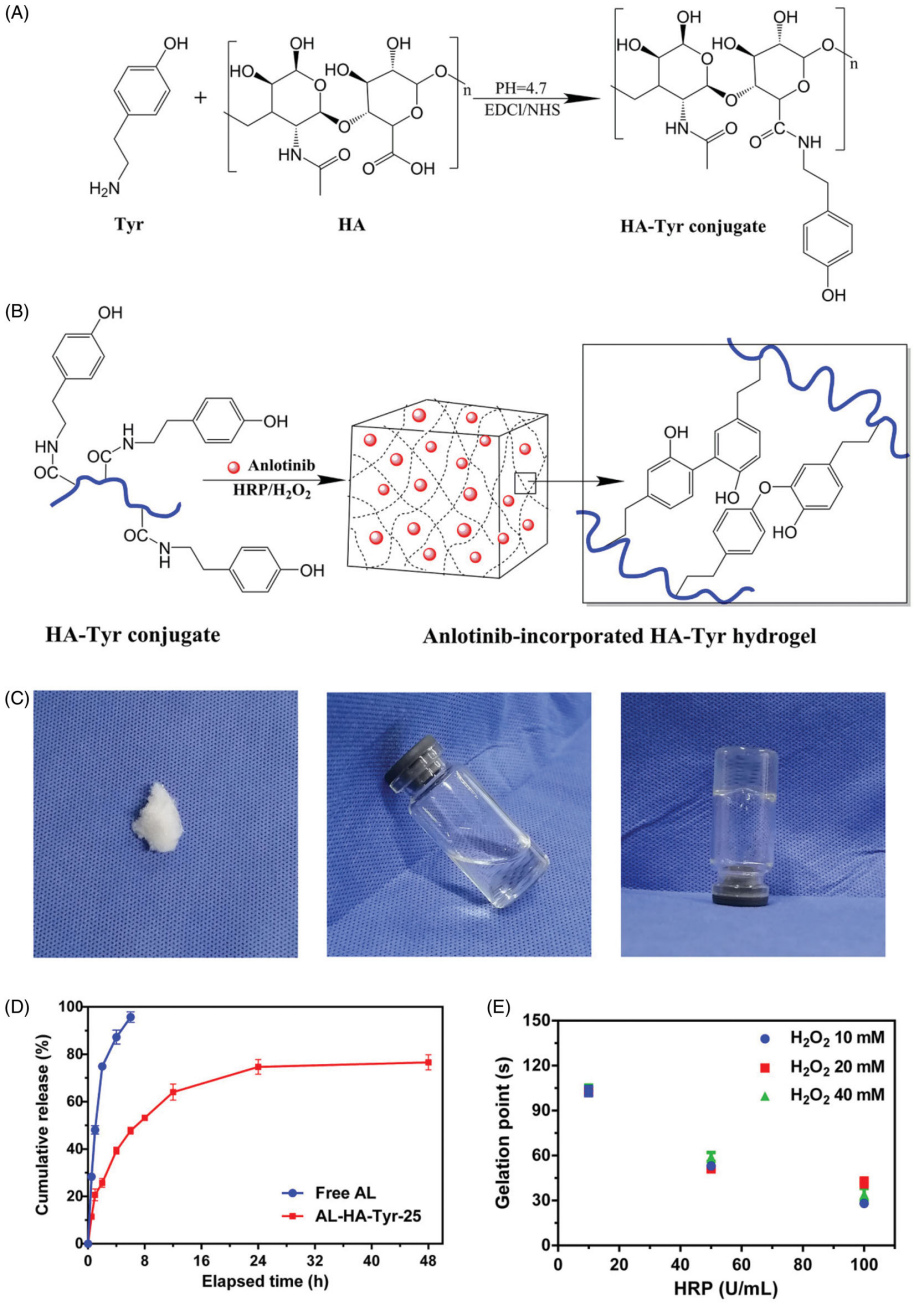
Synthesis and biochemical characteristics of AL–HA–Tyr hydrogels. (A) Synthesis of tyramine functionalized HA (HA–Tyr). (B) AL–HA–Tyr hydrogel formation. (C) Characterization of HA–Tyr hydrogel. Left: morphology of HA–Tyr after lyophilization. Center: HA dissolved in distilled water. Right: HA–Tyr hydrogel formation catalyzed by HPR and H_2_O_2_. (D) Cumulative release of AL from free AL, and AL–HA–Tyr + lysozyme-25 (*n* = 3). (E) Factors affecting gel formation. Gelation points of HA–Tyr conjugates at different concentration of H_2_O_2_ and HRP (*n* = 6). All data are shown as means ± SD.

Figure 1(A) shows the synthesis steps of AL–HA–Tyr. Conjugates HA–Tyr were synthesized under EDC·HCl/NHS activation. The ^1^H NMR results showed that the calculated degrees to which methyl groups in HA were substituted with tyramine (number of tyramine groups/100 HA repeat) was 18. Figure 1(C) shows the characteristics of the HA–Tyr hydrogel. Lyophilized HA–Tyr was white and flocculent (left panel) and became colorless, transparent, and fluid when dissolved in distilled water (1.0 wt.%) (center panel). When mixed with HRP (50 U/mL) and H_2_O_2_ (20 mM), HA–Tyr became colorless, transparent, non-fluid, and semi-solid (right panel). The AL–HA–Tyr hydrogel was formed via oxidative coupling of tyramine moieties catalyzed by H_2_O_2_ and HRP (Figure 1(B)).

The phase transition was determined by tube inversion. Figure 1(E) shows that under a fixed concentration of H_2_O_2_ and HRP concentrations of 10, 50, and 100 U/mL, the HA–Tyr conjugate gelled at 100, 50, and 30 s, respectively. When the concentration of HRP remained constant and that of H_2_O_2_ was changed, the gelation time remained the same. Thus, the gelation rate of the hydrogel can be controlled by changing the HRP concentration in the catalytic milieu of HRP/H_2_O_2_. *In vitro* release curves of AL–HA–Tyr-25 were assessed (Figure 1(D)). AL was released from the AL–HA–Tyr hydrogel by lysozyme over an extended period. Within 8 h, 95.7% of free AL in the dialysis bag was released into the dialysate. The AL–HA–Tyr-25 release rate was ∼80% over 48 h. These findings indicate the potential of HA–Tyr conjugates to increase therapeutic drug accumulation in target areas.

### 3.2. Inhibitory effect of AL–HA–Tyr

**Figure 2.**
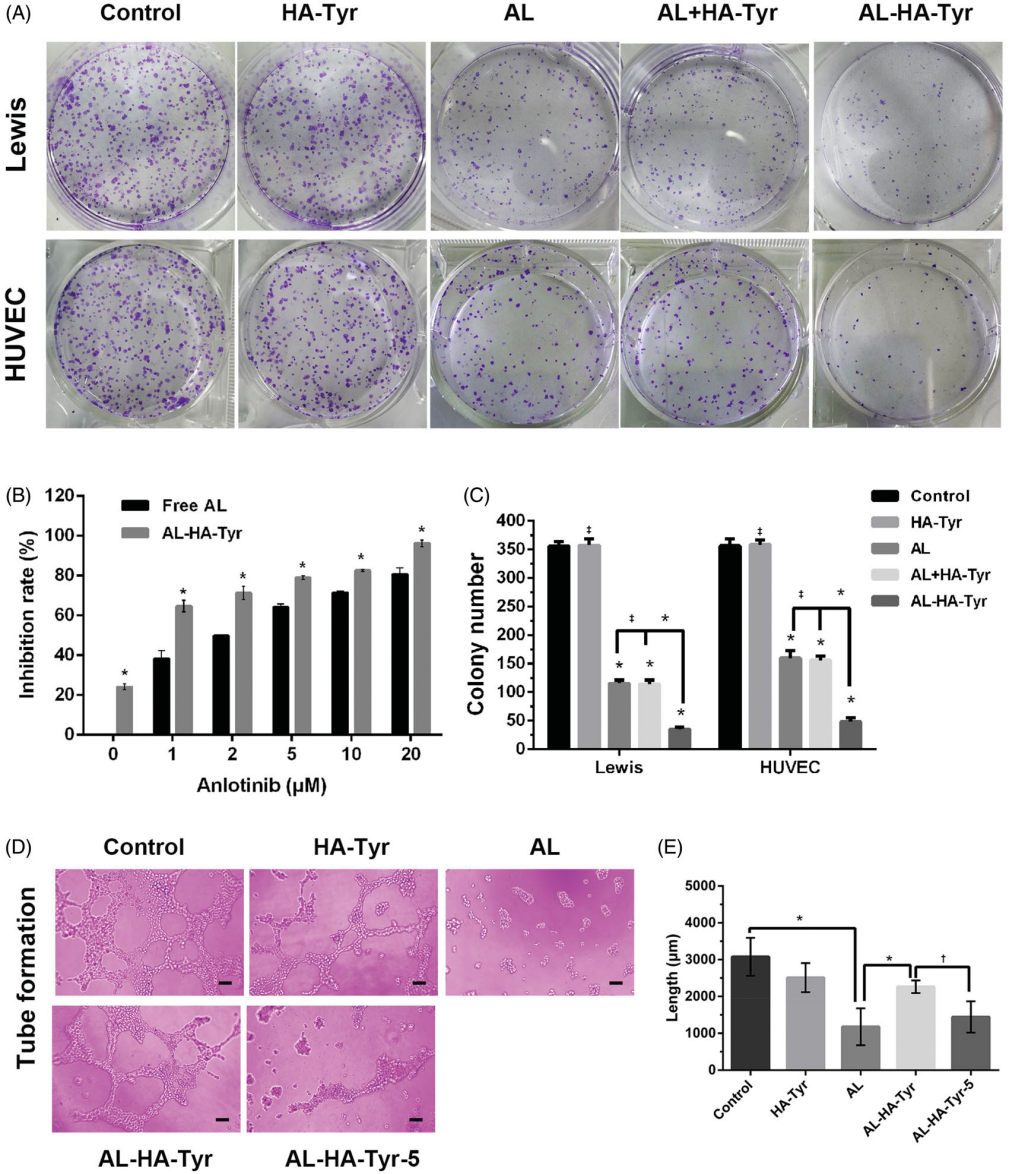
Effects of AL–HA–Tyr on colony formation and tubule formation. (A) Representative images of colony formation assays using Lewis lung cancer cells and HUVECs incubated with NS, HA–Tyr, AL, AL + HA–Tyr, or AL–HA–Tyr + lysozyme-5. (B) Rates of AL and AL–HA–Tyr + lysozyme-5 inhibition of HUVEC proliferation *in vitro.* (C) Colony number of HUVECs and Lewis lung cancer cells. Data are shown as the means ± SD (*n* = 3). *p<.01, †*p*<.05, and ‡*p*>.05. (D) HUVEC tubule formation after incubation with NS, HA–Tyr, AL, AL–HA–Tyr, or AL–HA–Tyr + lysozyme-5. (F) Total lengths of formed tubules. Data are shown as the means ± SD (*n* = 6). *p<.01, †*p*<.05, and ‡*p*>.05. Scale bar = 100 μm (D).

The rate of AL–HA–Tyr-5 inhibition of HUVECs and LLCs was evaluated in colony formation assays (Figure 2(A)). The inhibition rates of 0, 1, and 20 µM AL were 0%, 40%, and 80%, respectively. The inhibition rates of 0 and 20 µM AL–HA–Tyr-5 were ∼25% and ∼100%, respectively. Overall, AL–HA–Tyr-5 inhibited HUVEC colony formation more effectively than AL (*p*<.05). From the perspective of colony formation inhibition of LLCs and HUVEC after treatment with NS, HA–Tyr, AL, AL + HA–Tyr, or AL–HA–Tyr, there was no difference between HA and NS or AL and AL + HA–Tyr, indicating that HA–Tyr does not affect colony formation, whereas AL–HA–Tyr enhances colony formation inhibition, with consistent results observed between the two cell types.

### 3.3. AL–HA–Tyr inhibited HUVEC tubular formation

Figure 2(D) shows that AL exerted the maximum inhibitory effects on HUVEC tubular formation. Both AL (1178 ± 203.6) and AL–HA–Tyr-5 (1444 ± 173.4) significantly inhibited the formation of endothelial cell tubules (Figure 2(E)), whereas AL–HA–Tyr (2263 ± 69.75) was somewhat inhibitory. The tube length was calculated using ImageJ software (NIH, Bethesda, MD). The control (3077 ± 211.5) significantly differed from AL, AL and AL–HA–Tyr, and AL–HA–Tyr and AL–HA–Tyr-5 (*p*<.005 for all).

### 3.4. Al-HA–Tyr inhibited HUVEC and LLC cell migration and invasion

**Figure 3.**
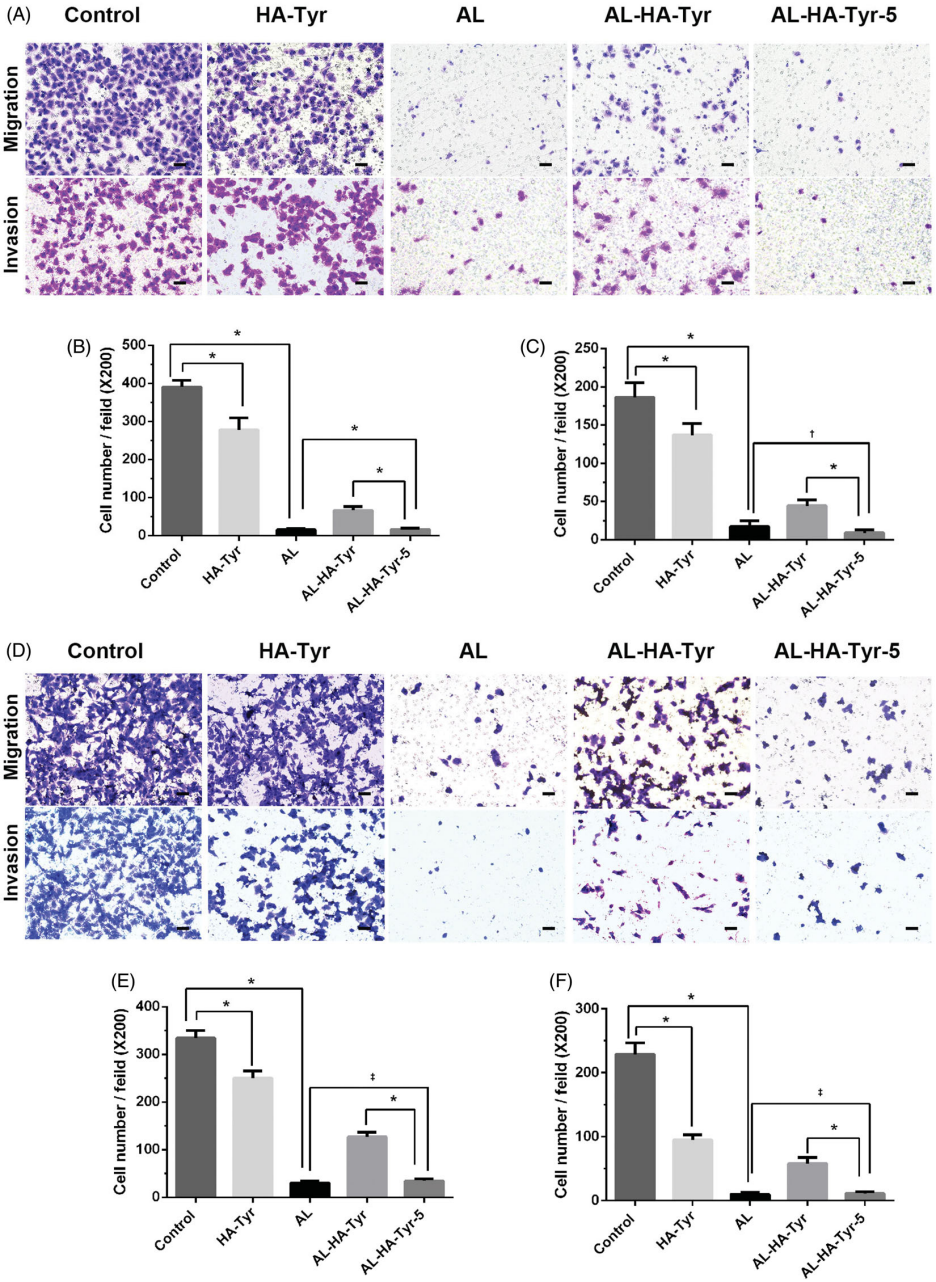
Inhibition of HUVEC and Lewis lung cancer cell migration and invasion. (A, D) Inhibition after incubation with NS, HA–Tyr, AL, AL–HA–Tyr, and AL–HA–Tyr-5. (B, C, E, F) Cell number of migrating and invading cells. Data are shown as the means ± SD (*n* = 6). *p<.01, †*p*<.05, and ‡*p*>.05. Scale bar = 50 μm.

The effects of AL–HA–Tyr on HUVEC and LLC cell migration and invasion were examined in transwell^®^ assays. Figure 3 shows that AL exerted the maximum inhibitory effects on HUVEC and LLC cell migration and invasion. The migration rates of HUVEC cells were 15.3 ± 1.5 and 16.3 ± 1.4 for AL and AL–HA–Tyr-5, respectively (*p*>.05). AL–HA–Tyr-5 and AL inhibited HUVEC invasion at rates of 8.8 ± 1.6 and 17.3 ± 3.1, respectively (*p*<.05). The migration rates of LLC cells were 30.3 ± 1.2 and 34.5 ± 1.8 for AL and AL–HA–Tyr-5, respectively (*p*>.05). AL–HA–Tyr-5 and AL inhibited LLC cell invasion at rates of 9.8 ± 1.0 and 11.3 ± 1.1, respectively (*p*>.05). The invasion and migration cells decreased in the order of control, HA–Tyr, AL–HA–Tyr, AL, and AL–HA–Tyr-5. The differences between AL–HA–Tyr and AL–HA–Tyr-5 (*p*<.005) were due to lysozyme action.

### 3.5. AL–HA–Tyr inhibited HUVEC and LLC cell-cycle and cell apoptosis

**Figure 4.**
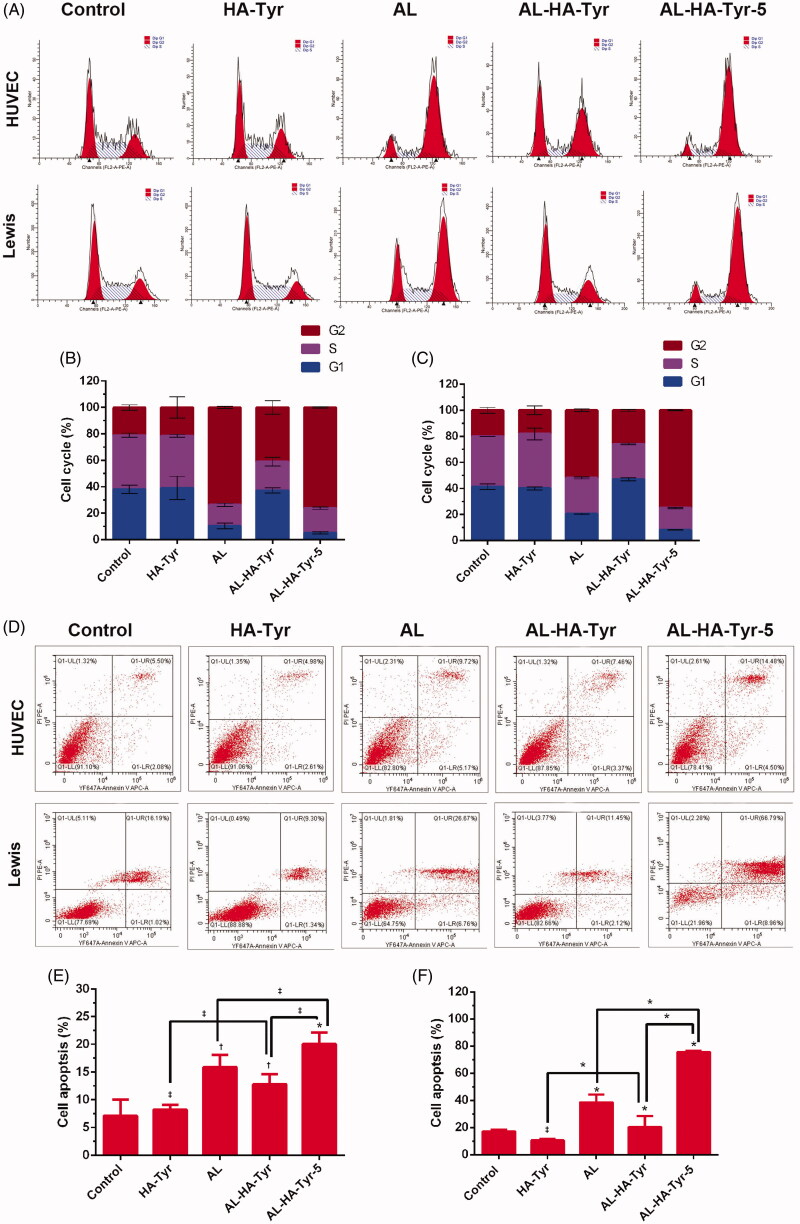
Analysis cell-cycle distribution and cell apoptosis of HUVECs and Lewis lung cancer cells. (A) Cell-cycle distribution and (D) cell apoptosis after incubation with NS, HA–Tyr, AL, AL–HA–Tyr, and AL–HA–Tyr-5; (B, C) quantitative results of cells in G1, S, and G2 phase; (E, F) percentage of apoptosis cells in various groups. Data are shown as the means ± SD (*n* = 6). *p<.01, †*p*<.05, and ‡*p*>.05. LL, LR, and UR represent living cells, early apoptotic cells, and late apoptotic cells or dead cells, respectively.

Figure 4(A) shows a representative image of the percentage of cells in each group at different stages of the cell cycle. Figure 4(B,C) shows a quantitative analysis of the cell cycle. The percentage of G1 phase cells in HUVECs after treatment with AL–HA–Tyr-5 (5.1 ± 0.4%) was significantly lower than that after treatment with AL (10.5 ± 1.2%), AL–HA–Tyr (37.2 ± 1.2), control (38.1 ± 1.7%), and HA–Tyr (39.2 ± 5.0%) (*p*<.05). The percentage of G1 phase LLC cells treated with AL–HA–Tyr-5 (8.1 ± 0.1%) was significantly lower than those treated with AL (20.5 ± 2.0%) and HA–Tyr (40.1 ± 0.6%), control (41.4 ± 1.3%) and AL–HA–Tyr (46.9 ± 0.7) (*p*<.05). There was no difference between the HA–Tyr and control groups (*p*>.05). Figure 4(D) shows a representative image of cell apoptosis. The percentage of apoptotic cells is shown in Figure 4(E,F): HA–Tyr had no significant effect on HUVEC and LLC cell apoptosis compared with controls (*p*>.05). The mortality rate (UR + LR) of LLC cells treated with AL–HA–Tyr-5 (75.5 ± 0.4%) was significantly higher than that of cells treated with AL (38.43 ± 3.4) (*p*<.01). There was no significant difference in the mortality (UR + LR) of HUVECs between treatment with AL–HA–Tyr-5 (20.0 ± 1.2%) and AL (15.92 ± 1.3%) (*p*<.05). AL had little effect on HUVEC apoptosis compared to that of LLC cells.

### 3.6. AL–HA–Tyr enhanced antitumor effects and prolonged survival

**Figure 5.**
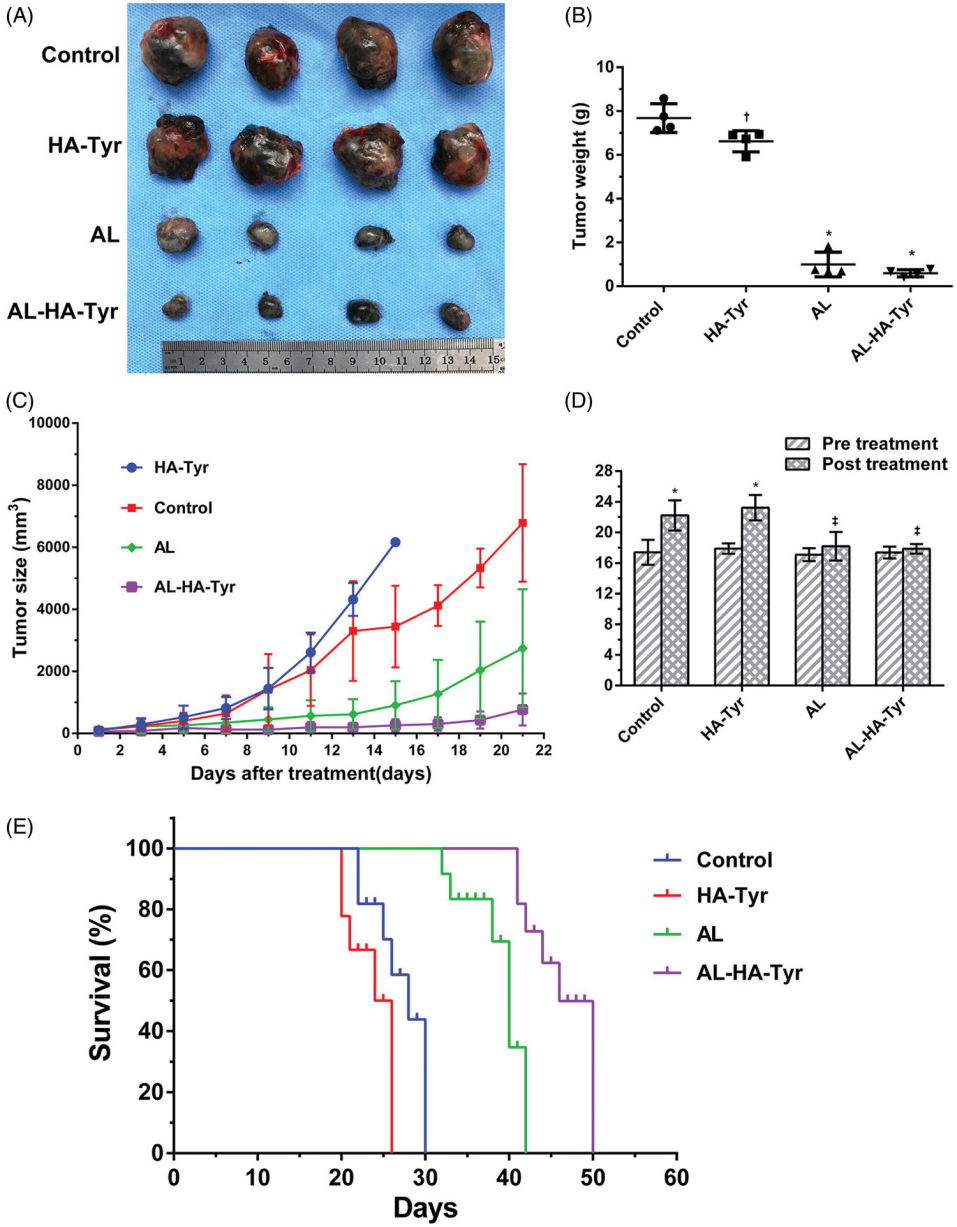
Tumor growth was inhibited and survival was prolonged by AL–HA–Tyr in LLC model. (A) Gross image of representative tumors excised from sacrificed mice. (B) Weight of excised tumors (*n* = 4; *p<.01, control vs. HA–Tyr, AL, and AL–HA–Tyr. (C) Tumor size at indicated times (*n* = 6). (D) Changes in weight of mice pre- and post-treatment (*n* = 12). *p<.01, †*p*<.05, ‡*p*>.05 pretreatment compared to post-treatment. (E) Survival of mice in each group (*n* = 6).

Figure 5(A,B) shows the general tumor volume and weight of mice after treatment. Tumors in control mice and those treated with HA–Tyr increased significantly, whereas those treated with AL and AL–HA–Tyr increased slightly. The control group had the largest tumors, followed by the HA–Tyr and AL and AL–HA–Tyr groups (Figure 5(B)); tumor masses were 13-fold higher in control mice than in those treated with AL–HA–Tyr (7.680 ± 0.3287 vs. 0.5950 ± 0.08150), showing that AL–HA–Tyr significantly inhibited tumor growth (*p* *<* .005). Treatment was stopped after 14 days, and tumor volume was measured at seven days thereafter (Figure 5(C)). The growth of tumors rapidly increased in the order of HA, control, AL, and AL–HA–Tyr. Tumors began to grow slightly at five days after AL–HA–Tyr injection.

Body weight differed significantly between the control and HA–Tyr groups before and after treatment (Figure 5(D)). The weight of control and HA–Tyr mice increased by 27.58% and 29.6%, respectively (*p*<.01), and was associated with rapid tumor growth that accounted for 34.59% and 28.53% of their body weight, respectively. Although body weight increased, the mice became emaciated and weak. Body weight did not significantly change in the AL and AL–HA–Tyr groups (*p*>.05).

AL–HA–Tyr significantly prolonged survival by 20 days (Figure 5(E)). The median survival times of the AL–HA–Tyr and AL groups were 46 and 37 days, respectively, compared with those of the HA–Tyr and control groups, which were 23 and 26 days, respectively. Overall, AL–HA–Tyr treatment significantly prolonged survival.

### 3.7. Decrease in visceral toxicity and Ki-67 and VEGF-A level by AL–HA–Tyr

**Figure 6.**
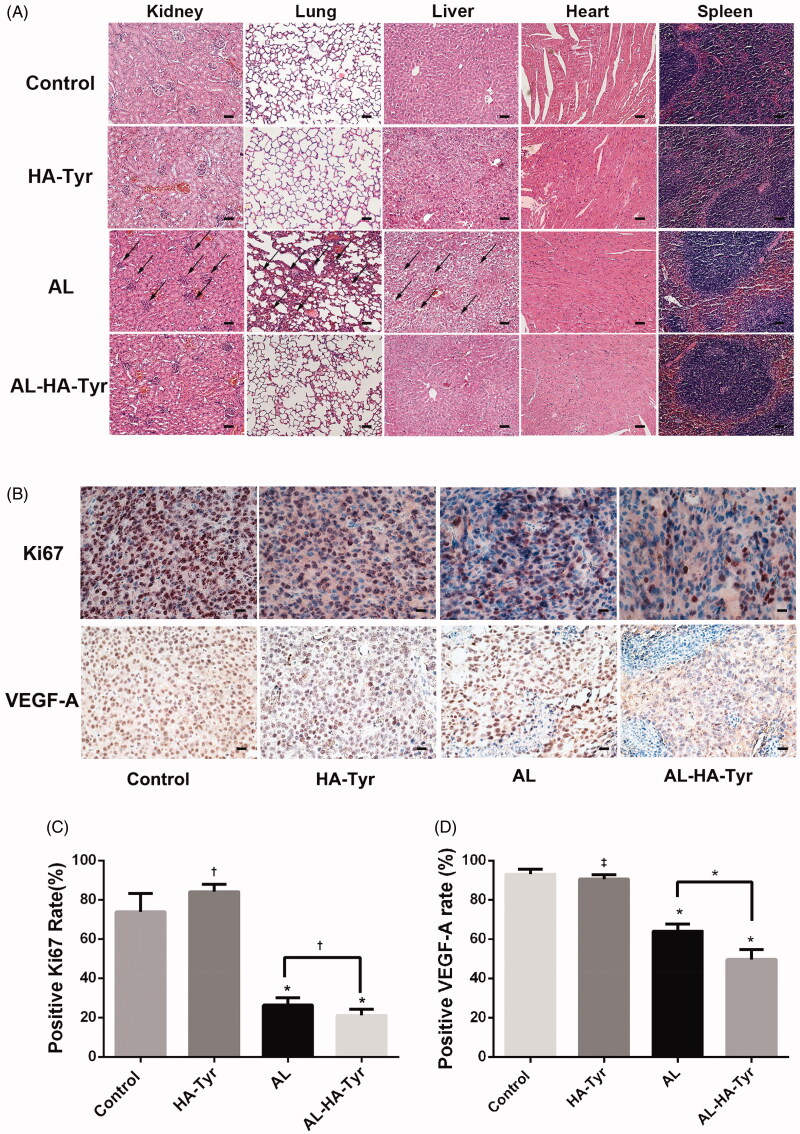
Histopathology and immunohistochemistry of organs in Lewis lung cancer cell tumor-bearing mice. (A) Toxicity was evaluated by HE staining of visceral tissues. Atrophy of renal corpuscles, proliferation of alveolar cells, and ballooning degeneration of hepatocytes are indicated by black arrows. Scale bar = 50 μm. (B) Representative immunohistochemical images show Ki-67 and VEGF-A expression in tumor tissues. Scale bar = 20 μm. (C, D) Ratios (%) of Ki-67-positive cells and VEGF-A-positive cells in each group. Data are expressed as the means ± SD. †*p*<.05, *p<.01, ‡*p*>.05, HA–Tyr, AL, and AL–HA–Tyr vs. control; AL–HA–Tyr vs. AL.

Toxicity was evaluated in HE-stained visceral tissues (Figure 6(A)). The features of the heart and spleen were the same in each group, whereas they differed in the kidneys, lungs, and livers of different groups. The morphology of visceral tissues was normal in the control and HA–Tyr groups. However, treatment with AL resulted in a large amount of atrophic renal corpuscles, absent renal vesicles leaving only glomeruli in the kidneys, obvious proliferation of alveolar cells in the lungs, and balloon-like degeneration of hepatocytes in the liver. Although renal corpuscles were slightly atrophic, the structure was unchanged; moreover, the lungs and liver were essentially normal in the AL–HA–Tyr group. These findings show that AL–HA–Tyr reduced visceral toxicity in mice.

Furthermore, AL–HA–Tyr decreased the proliferation index of tumor cells as evaluated by Ki-67 expression (Figure 6(C)). The relative proportion of Ki-67-positive cells significantly decreased in AL–HA–Tyr vs. AL, HA–Tyr, and control mice (21.17 ± 1.249%, 26.33 ± 1.563%, 84.17 ± 1.537%, and 73.83 ± 3.877%, respectively). Staining with Ki-67 was denser in the AL than in the AL–HA–Tyr group (*p*<.05; Figure 6(C)).

AL–HA–Tyr decreased VEGF-A expression in tumor cells (Figure 6(D)). The relative proportion of VEGF-A-positive cells significantly decreased in the AL–HA–Tyr vs. AL, HA–Tyr, and control mice (49.67 ± 2.06%, 64.17 ± 1.42%, 90.5 ± 0.88%, and 93.17 ± 1.01%, respectively). VEGF-A expression in tumor cells significantly differed between mice in the AL and AL–HA–Tyr groups (*p*<.05; Figure 6(D)).

## 4. Discussion

Lung cancer accounts for the highest morbidity worldwide among cancer types (Bray et al., 2018). CT-guided percutaneous needle biopsy has become an essential method for pathological verification (Tongbai et al., 2019), providing a technical background for applying intratumoral injection with the potential to deliver drugs in a bolus. Anti-angiogenesis therapy is an effective treatment strategy. AL, a novel small molecule inhibitor of multiple receptor Tyr kinases, suppresses neoplastic angiogenesis and tumor growth and is effective against solid tumors (He et al., 2018; Lin et al., 2018; Ruan et al., 2019; Wang et al., 2019). It was approved as an optional third-line treatment for refractory advanced non-small cell lung cancer (Han et al., 2018a,b; Chen, 2019; Zhou et al., 2019). Like other analogous agents, the most common adverse effects of AL are hypertension, triglyceride elevation, elevated thyroid-stimulating hormone (TSH) (Sun et al., 2016; Si et al., 2018; Zhao et al., 2018). The pattern of systemic administration determines the increase in local blood concentrations along with increased systemic cardiovascular toxicity. Therefore, a mode of local drug delivery that directly acts on tumors is needed to reduce toxicity. HA and its derivatives have been widely used because of their biocompatibility and biodegradability. An injectable and biodegradable hydrogel formed by HA and Tyr and its sustained release and safety features have also been confirmed (Kurisawa et al., 2005).

We found that AL can be encapsulated in a HA–Tyr hydrogel (AL–HA–Tyr) in an acidic environment. A white floc appeared over time when it was dissolved in PBS at pH 7.4, which may be associated with the pH-dependent hydrophilicity of AL (Zhong et al., 2018). We changed the conditions of AL–HA–Tyr synthesis and achieved continuous drug release *in vitro*. The uptake of antiangiogenic drugs in solid tumors is extremely low in tumor-bearing mice, whereas it is higher in visceral organs. Compared with AL, AL–HA–Tyr reduced toxicity to the lungs, liver, and kidneys of C57BL/6J mice. These findings are consistent with those of previous studies indicating that HA forms of drugs such as paclitaxel and doxorubicin have better curative effects and lower toxicity (Saravanakumar et al., 2010; Yoon et al., 2012; Zhao et al., 2017; Liang et al., 2018). Tumor angiogenesis has been attributed to increased tumor growth and metastatic potential; thus, angiogenesis inhibition is an attractive strategy for treating cancer (Paduch, 2016). We showed that AL–HA–Tyr-5 enhanced the inhibition of colony formation of HUVECs and LLC cells and reduced the numbers of surviving colonies *in vitro* in a dose-dependent manner. HA–Tyr had no effect on colony formation but somewhat inhibited cell migration and invasion and tube formation. This may be because HA is a biological macromolecule that shows high viscosity in aqueous solution. Therefore, the effect of HA–Tyr on migration and invasion may be related to the viscosity of the culture medium. AL–HA–Tyr-5 inhibited not only angiogenesis, but also migration and invasion. However, AL–HA–Tyr-5 had less inhibitory effects on tube formation than AL because of its incomplete release. In the analysis of cell cycle and apoptosis, AL–HA–Tyr inhibited the cell cycle, had little effect on HUVEC apoptosis, had a large impact on LLC cell apoptosis, and showed direct anti-tumor effects. We also assessed its antitumor effects *in vivo* by detecting Ki67 expression and anti-angiogenesis effects by detecting VEGF-A. We found that AL–HA–Tyr significantly reduced the ratios of Ki67- and VEGF-A positive cells in transplanted tumors and significantly prolonged the survival of tumor-bearing mice. As reported in other studies, HA–Tyr hydrogels enhanced therapeutic effectiveness and allowed for continuous release (Xu et al., 2013, 2015; Ueda et al., 2016; Tang et al., 2019).

In this study, AL–HA–Tyr release was measured in a simulated environment *in vivo*, whereas sustained release may be affected in the real tumor microenvironment. We synthesized AL–HA–Tyr and verified its effectiveness and safety in LLC tumor-bearing mice. Whether it is equally effective against other tumors remains unknown and requires further investigation. Additionally, we did not use an orthotopic model that because the establishment of orthotropic model and tumor formation, measurement, detection, and administration remain difficult, particularly administration; further, the trauma may be too great for mice, which may lead to death and affect the results. As a preliminary observation of the efficacy of AL–HA–Tyr, the xenograft model makes it easier to observe tumor growth, measure tumor size, and administer drugs. Overall, AL–HA–Tyr offers controlled release, along with antitumor and antiangiogenic properties. AL–HA–Tyr may be suitable for delivering anticancer agents to other types of solid tumors, as AL has broad-spectrum antitumor effects. Therefore, we used AL–HA–Tyr hydrogel after biopsy to block the needle channel, reduce the complications of biopsies, and provide an early intervention, indicating that it can be adapted for various practical applications.

In conclusion, AL was incorporated into an injectable HA–Tyr hydrogel and controlled release was achieved. The conjugate, AL–HA–Tyr, exerted better antitumor and antiangiogenic effects with lower toxicity following intratumoral administration and significantly prolonged the survival of mice bearing LLC tumors.
